# Protease-activated receptor-1 deficiency protects against streptozotocin-induced diabetic nephropathy in mice

**DOI:** 10.1038/srep33030

**Published:** 2016-09-13

**Authors:** Maaike Waasdorp, JanWillem Duitman, Sandrine Florquin, C. Arnold Spek

**Affiliations:** 1 Center for Experimental and Molecular Medicine, Academic Medical Center, Amsterdam, 1105 AZ, The Netherlands; 2 Department of Pathology, Academic Medical Center, Amsterdam, 1105 AZ, The Netherlands

## Abstract

Endogenously administered activated protein C ameliorates diabetic nephropathy (DN) in a protease-activated receptor-1 (PAR-1)-dependent manner, suggesting that PAR-1 activation limits the progression of DN. Activation of PAR-1 in fibroblast-like cells, however, induces proliferation and extracellular matrix production, thereby driving fibrotic disease. Considering the key role of mesangial proliferation and extracellular matrix production during DN, PAR-1 may in fact potentiate diabetes-induced kidney injury. To determine the net effect of PAR-1 in DN, streptozotocin-induced DN was studied in wild type and PAR-1 deficient mice. Subsequent mechanistic insight was obtained by assessing profibrotic responses of mesangial and tubular epithelial cells *in vitro*, following PAR-1 stimulation and inhibition. Despite having similar glucose levels, PAR-1 deficient mice developed less kidney damage after induction of diabetes, as evidenced by diminished proteinuria, plasma cystatin C levels, expansion of the mesangial area, and tubular atrophy. *In vitro*, PAR-1 signaling in mesangial cells led to increased proliferation and expression of matrix proteins fibronectin and collagen IV. Conversely, a reduction in both proliferation and fibronectin deposition was observed in diabetic PAR-1 deficient mice. Overall, we show that PAR-1 plays an important role in the development of DN and PAR-1 might therefore be an attractive therapeutic target to pursue in DN.

Diabetic nephropathy is one of the most important complications of diabetes mellitus. Currently, around 30% of diabetic patients develop nephropathy, but the incidence is rapidly rising, as a result of the growing number of patients suffering from type II diabetes, in combination with the earlier onset of the disease^1^. Treatment options are limited, and although the progression of diabetic nephropathy may be delayed by strict control of the plasma glucose concentration and/or by lowering the blood pressure, the majority of patients will eventually progress to end-stage renal disease (ESRD)^2^. Once being diagnosed with this life threatening condition, renal replacement therapy, either by dialysis or kidney transplantation, remains the last resort. As both options are a huge burden to patients and society, alternative (preventive) treatment options are eagerly awaited for. Consequently, better insight into the molecular pathogenesis of diabetic nephropathy is warranted.

Pathologically, diabetic nephropathy is a fibroproliferative disease, characterized by glomerular hypertrophy due to proliferation and excessive matrix production of mesangial cells^3^. Interestingly, protease-activated receptor (PAR)-1 has recently been identified as a key player in fibroproliferative diseases. Indeed, PAR-1 drives fibroblast proliferation and extracellular matrix production *in vitro*^4^, whereas PAR-1 deficiency limits liver^5^, lung^6^ and skin^7^ fibrosis in experimental animal models.

PAR-1 is a seven transmembrane domain receptor that belongs to the family of G protein-coupled receptors (GPCRs)^8^. As opposed to classical GPCRs, PAR-1 activation requires proteolytic cleavage rather than ligand binding. PAR-1 was originally identified as the thrombin receptor^8^ although recently other agonists, such as activated protein C (APC), have been described^9^,^10^. Proteolytic removal of the N-terminal extracellular region of PAR-1 by these agonists releases a newly tethered ligand that interacts with the body of the receptor to induce transmembrane signaling thereby triggering a broad range of signaling pathways and affecting multiple pathophysiological responses^9^.

In the kidney, PAR-1 is expressed in the mesangial cells of the glomeruli and, based on the key role of PAR-1 in fibroproliferative disease, it is tempting to speculate that PAR-1 may be a key factor driving the pathogenesis of diabetic nephropathy. In line with this hypothesis, tubular expression of PAR-1 is markedly up-regulated in patients with chronic allograft nephropathy, and PAR-1 levels correlate with interstitial fibrin deposition, the degree of tubulointerstitial fibrosis, and urinary excretion of transforming growth factor-beta^11^. Moreover, PAR-1 is upregulated in glomeruli of diabetic mice, and the use of PAR-1 knockout mice effectively reduced crescent formation, glomerular inflammatory cell infiltration, and serum creatinine concentrations in a murine model of crescentic glomerulonephritis^12^. Interestingly, however, APC was recently shown to protect mice against diabetic nephropathy. In line with these findings, APC prevented hyperglycemia-induced apoptosis of endothelial cells and podocytes in a PAR-1 dependent manner *in vitro*^13^, suggesting that PAR-1 may play a nephroprotective role during diabetic nephropathy. Although PAR-1 seems to orchestrate fibroproliferative responses in renal disease, its actual importance and net effect in diabetic nephropathy remains elusive.

In the present study, we aimed to elucidate the net effect of PAR-1 during the development of diabetic nephropathy by subjecting wild type and PAR-1 deficient mice to a well-established model of streptozotocin-induced diabetes. We show that the absence of PAR-1 affords protection against diabetes-induced nephropathy, and we hypothesize that PAR-1 drives diabetic nephropathy mainly by inducing mesangial cell proliferation and extracellular matrix production.

## Results

### Diabetic nephropathy is reduced in PAR-1 deficient mice

**Figure 1:**
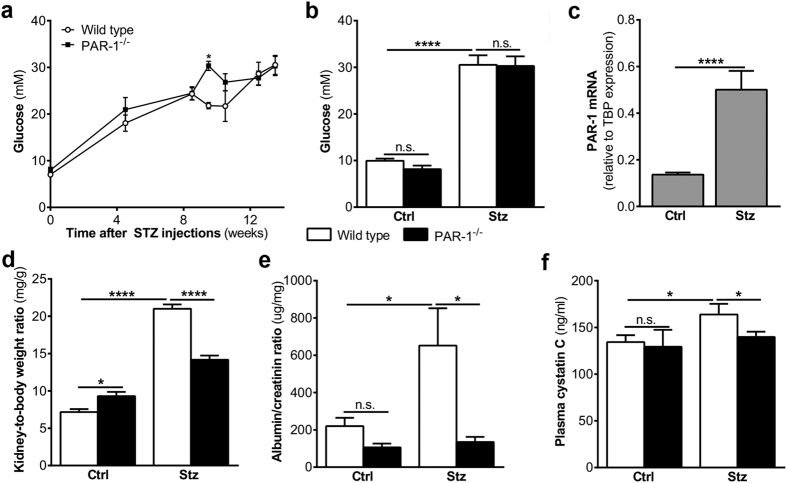
Reduced nephropathy in PAR-1 deficient diabetic mice. (**a**) Glucose levels of wild type and PAR-1 deficient mice during the experiment. (**b**) Glucose levels of wild type and PAR-1 deficient mice three months after streptozotocin (stz) or saline (ctrl) injections. (**c**) PAR-1 mRNA expression in mouse kidney homogenates of Wt mice three months after streptozotocine (stz) or saline (ctrl) injections. TBP expression served as reference gene. (**d**) Kidney-to-body weight ratio, (**e**) urinary albumin/creatinine ration, and (**f**) plasma cystatin C levels of wild type and PAR-1 deficient mice three months after streptozotocin (stz) or saline (ctrl) injections. Indicated is the mean ± SEM, Wt ctrl, n = 10; PAR-1^−/−^ ctrl, n = 7; Wt stz, n = 6; PAR-1^−/−^ stz, n = 8. Kruskal Wallis with Dunns post-hoc analysis, one-way ANOVA with Bonferroni post-hoc analysis and unpaired t-test were used, *p < 0.05; ****p < 0.0001.

To assess the importance of PAR-1 during the development of diabetic nephropathy, wild type and PAR-1 deficient mice were subjected to an experimental model of streptozotocin-induced diabetes. As shown in Fig. 1a,b, both wild type and PAR-1 deficient mice developed diabetes, as was evident from the elevated glucose levels of approximately 30 mM, measured upon sacrifice. In wild type mice, hyperglycemia was associated with increased Par-1 mRNA levels in the kidneys (Fig. 1c). Persistent hyperglycemia induced nephropathy as evidenced by an increase in the kidney-to-body weight ratio, proteinuria and elevated plasma cystatin C levels in wild type mice (Fig. 1d–f). Interestingly, all nephropathy markers were significantly reduced in PAR-1 deficient mice as compared to wild type mice, suggesting that PAR-1 potentiates diabetic nephropathy.

**Figure 2:**
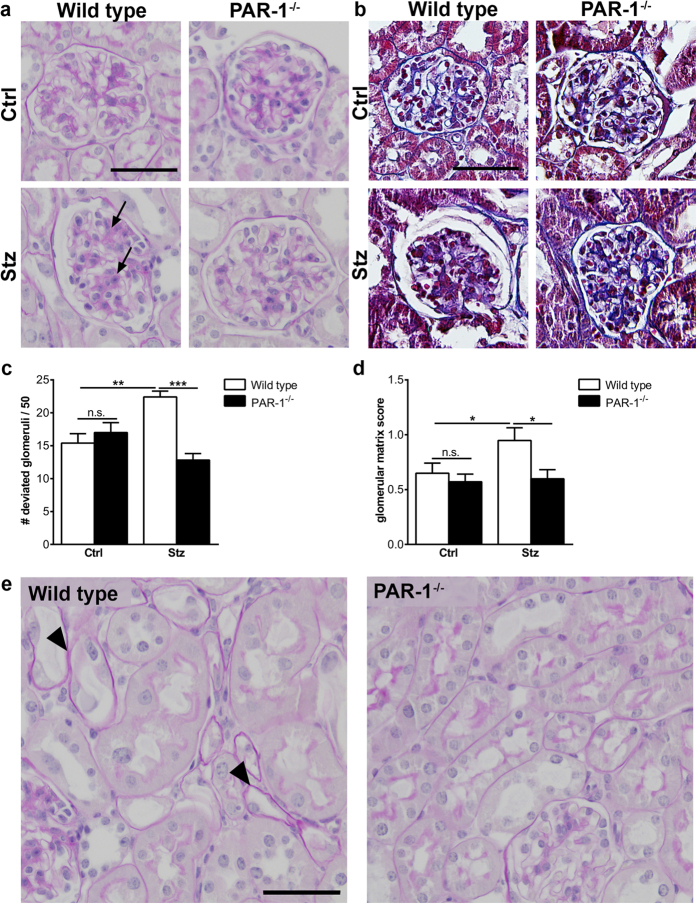
Mesangial expansion in PAR-1 deficient diabetic mice. (**a**) Representative pictures of PAS-D stained paraffin sections of kidneys of wild type and PAR-1 deficient mice, three months after streptozotocin (stz) or saline (ctrl) injections. Arrows pinpoint areas of mesangial expansion. Scale bar: 50 μm. (**b**) Masson’s Trichrome stained paraffin sections of kidneys of wild type and PAR-1 deficient mice three months after streptozotocin (stz) or saline (ctrl) injections. Scale bar: 50 μM. (**c**) Quantification of mesangial expansion in glomeruli of diabetic (stz) and non-diabetic (ctrl) wild type and PAR-1 deficient mice. Glomeruli were scored either normal or deviated as described in the materials and methods section. Wt ctrl, n = 5; PAR-1^−/−^ ctrl, n = 7; Wt stz, n = 6; PAR-1^−/−^ stz, n = 8. (**d**) Quantification of histological evaluation of glomerular extracellular matrix deposition. Indicated is the mean ± SEM, Wt ctrl, n = 5; PAR-1^−/−^ ctrl, n = 7; Wt stz, n = 5; PAR-1^−/−^ stz, n = 8. One outlier was excluded from analysis. (**e**) Representative picture of tubuli from diabetic wild type and PAR-1 deficient mice. Arrowheads pinpoint atrophic tubuli with flattening of the proximal tubular epithelial cells and thickening of the basement membrane. Scale bar: 50 μm. One-way ANOVA with Bonferroni post-hoc analysis and unpaired t-test were used, *p < 0.05; **p < 0.01; ***p < 0.005.

At the histological level, substantial expansion of the mesangial compartment was observed in about 50% of the glomeruli of diabetic wild type mice (illustrated in Fig. 2a and quantified in Fig. 2c). Strikingly, in PAR-1 deficient mice, only around 30% of the glomeruli showed mesangial expansion, which is comparable to age-matched non-diabetic control mice (Fig. 2c). In line with reduced mesangial expansion in PAR-1 deficient mice, mesangial matrix deposition, as visualized by Masson’s trichrome staining, was significantly reduced in PAR-1 deficient mice as compared to wild type mice (Fig. 2b,d). Moreover, tubular atrophy was observed in all diabetic wild type mice, whereas no tubular atrophy was observed in any of the PAR-1 deficient mice (Fig. 2e). Taken together, these data show that PAR-1 deficiency diminished diabetes-induced kidney damage.

### Mesangial expansion is reduced in diabetic PAR-1 deficient mice

**Figure 3:**
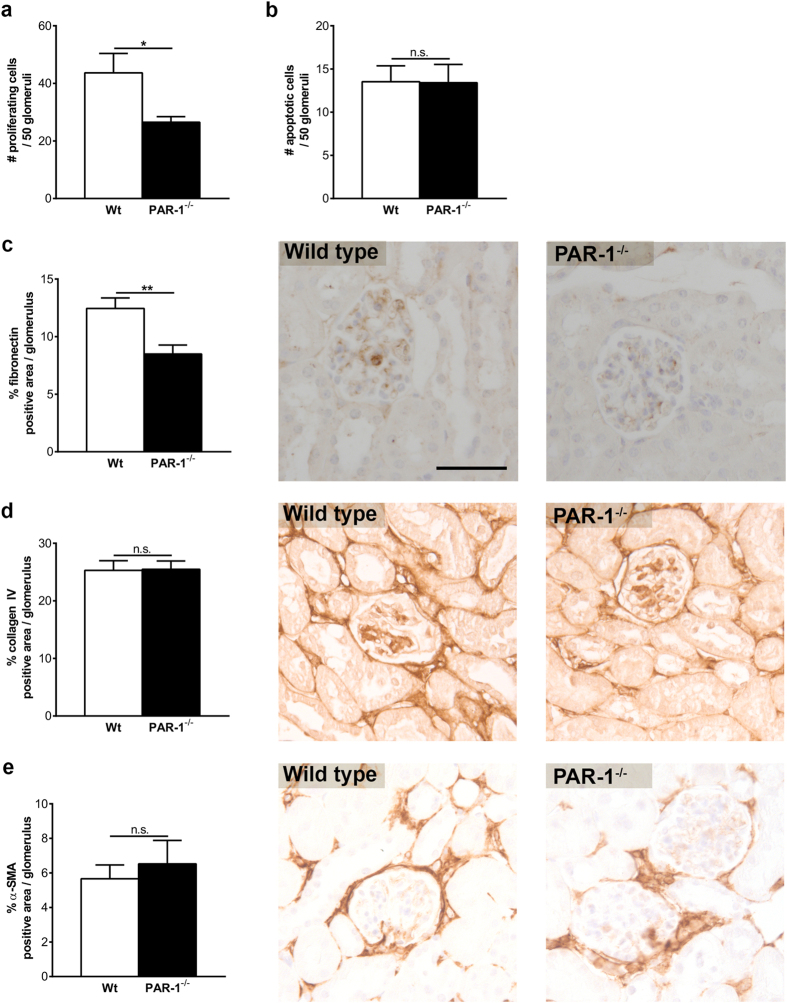
PAR-1 deficiency prevents excessive proliferation and extracellular matrix production *in vivo*. Paraffin sections obtained from wild type and PAR-1 deficient mice three months after streptozotocin injections were immunohistochemically stained (brown) and subsequently analysed. (**a**) The number of proliferating (Ki67 positive) cells per 50 glomeruli. (**b**) The number of apoptotic (cleaved caspase 3 positive) cells per 50 glomeruli. (**c–e**) Fibronectin (**c**), Collagen IV (**d**), and α-SMA (**e**) positive area per glomerulus. Quantification of the slides is shown in the left panel, representative examples are illustrated on the right. Scale bar (applies for all panels): 50 μm. Indicated is the mean ± SEM. Wt stz, n = 5; PAR-1^−/−^ stz, n = 8. One-sided unpaired t-tests were used for analysis. *p < 0.05; **p < 0.01.

To assess whether PAR-1 drives mesangial expansion during streptozotocin-induced diabetic nephropathy, we evaluated proliferation and apoptosis of mesangial cells in both diabetic wild type and PAR-1 deficient mice. As shown in Fig. 3a, the number of proliferating mesangial cells in the glomeruli of PAR-1 deficient mice was significantly decreased by about 50% compared to wild type mice. Interestingly, apoptosis was hardly observed in glomeruli of both diabetic wild type and PAR-1 deficient mice (Fig. 3b). The increased mesangial proliferation observed in wild type mice was not accompanied by alterations in collagen IV and α-SMA expression, whereas fibronectin deposition was significantly reduced in the glomeruli of PAR-1 deficient mice as compared to wild type mice (Fig. 3c–e). Together, these data show that PAR-1 deficiency limits diabetic nephropathy, at least in part, by preventing hyperglycemia-induced mesangial proliferation and fibronectin production.

### PAR-1 drives mesangial proliferation and extracellular matrix production *in vitro*

**Figure 4:**
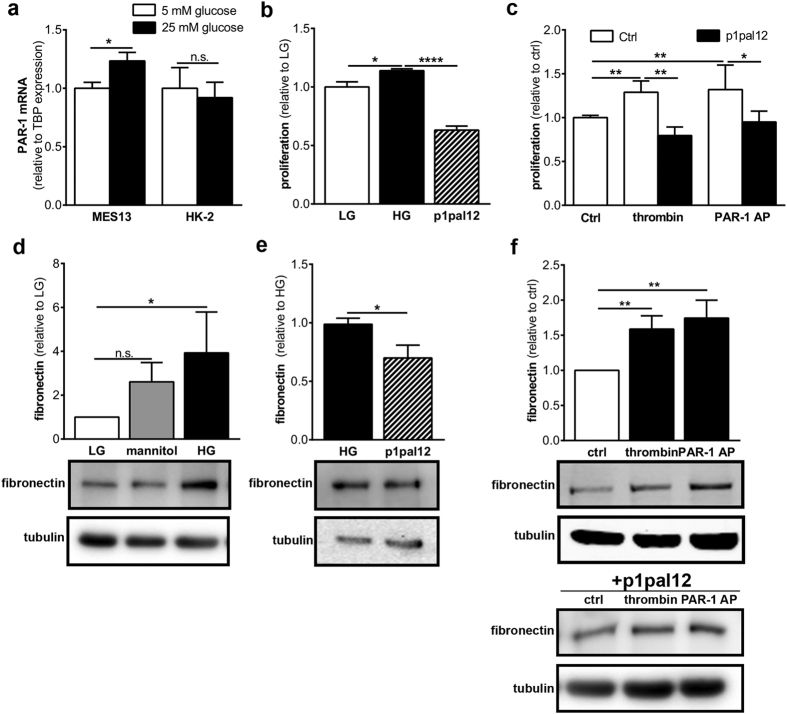
PAR-1 signaling leads to proliferation and extracellular matrix production MES13 cells. (**a**) PAR-1 mRNA expression in MES13 cells, HK-2 cells and imPTECs after 48 h of stimulation with 5 mM glucose (LG) or 25 mM glucose (HG). TBP expression served as reference gene. (**b**) Proliferation of MES13 cells after 24 h of stimulation with 25 mM glucose with and without p1pal12 (5 uM). (**c**) Proliferation of MES13 cells after 24 h stimulation with thrombin (1 U/ml) or PAR1-AP (100 μM) in the presence of p1pal12 (5 μM) or DMSO. (**d–f**) Western blot (lower panel) and quantification (upper panel) of fibronectin levels in MES13 whole cell lysates after 24 h stimulation with 20 mM mannitol or 25 mM glucose (**d**), p1pal12 (**e**), and thrombin (1 U/ml) or PAR1-AP (100 μM), with and without p1pal12 pretreatment (**f**). Indicated is the mean ± SEM, n = 3 individual experiments. One-way ANOVA with Bonferroni post-hoc analysis and unpaired t-test were used *p < 0.05; **p < 0.01; ****p < 0.01.

To assess whether PAR-1 activation directly drives mesangial expansion and/or tubular damage, we next assessed PAR-1 expression in MES13 and HK-2 cells. As shown in Fig. 4a, MES13 cells expressed PAR-1 under standard, low glucose, culture conditions, and PAR-1 levels increased in hyperglycemic culture conditions (25 mM glucose). HK-2 cells also expressed PAR-1 under standard conditions but PAR-1 was not significantly induced in these cells in hyperglycemic culture conditions (Fig. 4a). Hyperglycemia-induced PAR-1 expression in MES13 cells was accompanied by increased proliferation which was inhibited by pre-treatment with the specific PAR-1 inhibitor p1pal12 (Fig. 4b). Moreover, stimulation of MES13 cells with either thrombin or PAR-1 agonist peptide for 24 hours increased proliferation by approximately 35%. This increase was completely prevented by pre-treatment with p1pal12 (Fig. 4c). PAR-1 activation in HK-2 cells did not affect proliferation (Supplementary Fig. S1). In addition to inducing proliferation in MES13 cells, hyperglycemia also induced the production of the extracellular matrix protein fibronectin, whereas mannitol, a well-known osmotic control, did not (Fig. 4d). Hyperglycemia-induced fibronectin production was inhibited by P1pal12 (Fig. 4e). Moreover, PAR-1 stimulation with thrombin or PAR-1 agonist peptide led to increased production of fibronectin and, again, pre-treatment with p1pal-12 completely prevented thrombin or PAR-1 agonist peptide-induced fibronectin production (Fig. 4f).

**Figure 5:**
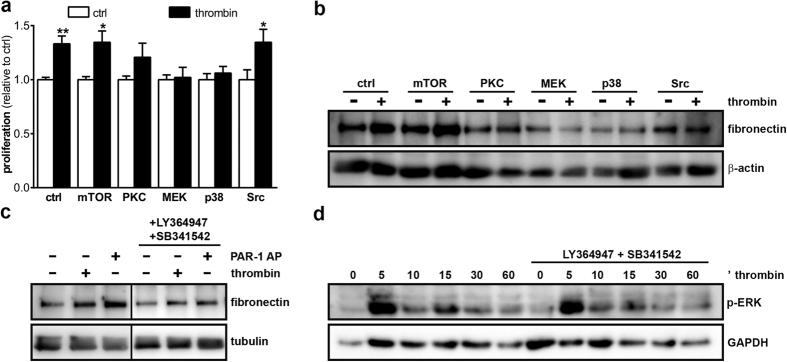
Thrombin induces proliferation and fibronectin production in a MEK-p38-PKC and MEK-p38-Src dependent manner. (**a**) Proliferation of MES13 cells after 24 h thrombin stimulation (1 U/ml) in the presence of inhibitors of mTOR (rapomycin, 20 nM), PKC (Chelerythrine, 1 μM), MEK (U0126, 10 μM), p38 (SB203580, 10 μM), or Src (PP1, 200 nM). (**b**) Western blot of fibronectin levels in MES13 whole cell lysates after 24 h thrombin stimulation in the presence of inhibitors of mTOR (rapomycin, 20 nM), PKC (Chelerythrine, 1 μM), MEK (U0126, 10 μM), p38 (SB203580, 10 μM), Src (PP1, 200 nM), or (**c**) TGFβ (10 μM LY364947 and 10 μM SB341542). (**d**) Western blot of phospho-ERK1/2 levels in MES13 whole cell lysates after thrombin stimulation for the indicated time points. Indicated is the mean ± SEM, n = 3 individual experiments. One-way ANOVA with Bonferroni post-hoc analysis and unpaired t-test were used *p < 0.05; **p < 0.01.

To assess the underlying mechanism by which PAR-1 stimulation induced proliferation and fibronectin production, we next assessed inhibition of key signaling pathways known to be involved in PAR-1 signaling. As shown in Fig. 5a, inhibition of MEK and p38 completely prevented PAR-1-induced proliferation, whereas PKC inhibition only marginally inhibited PAR-1-induced proliferation. On the contrary, both mTOR and Src inhibition did not affect PAR-1-induced proliferation. As shown in Fig. 5b, MEK, p38 and Src inhibition seem to limit PAR-1-induced fibronectin production whereas mTOR and PKC inhibition seems not to prevent PAR-1-induced fibronectin production. Moreover, TGFβ receptor inhibition also prevented PAR-1-induced fibronectin production (Fig. 5c), although both thrombin and PAR-1 agonist peptide did not induce TGFβ secretion by MES13 cells (data not shown). Finally, TGFβ receptor inhibition did not modify PAR-1-induced ERK1/2 phosphorylation (Fig. 5d). Overall, these results suggest that PAR-1 directly drives mesangial expansion *in vitro*.

## Discussion

Diabetic nephropathy is a frequently observed complication of diabetes which ultimately requires renal replacement therapy. Treatment options to delay or prevent the development of diabetic nephropathy are therefore eagerly awaited. As diabetic nephropathy is characterized by fibroproliferative lesions, we aimed to elucidate the relevance of PAR-1, a well-known inducer of fibroproliferative processes, during diabetic nephropathy. To this end, we subjected wild type and PAR-1 deficient mice to a well-established model of streptozotocin-induced diabetes and assessed the severity of diabetic nephropathy. We indeed show that genetic ablation of PAR-1 limits the development of diabetic nephropathy in streptozotocin-treated mice.

In the current *in vivo* study, PAR-1 expression increased 3–4 times in the kidneys of diabetic mice as compared to non-diabetic controls. Moreover, MES13 cells cultured in high glucose containing culture medium showed increased PAR-1 expression as compared to low glucose culture conditions. These data, which are in line with work published by Sakai *et al*., showing increased PAR-1 expression and specific glomerular localization of PAR-1 in a db/db model of diabetic nephropathy^14^, already suggest that PAR-1 might play a role during the development of diabetic nephropathy.

Interestingly, pharmacological treatment with APC limited diabetic nephropathy in a PAR-1 dependent manner in a similar model of streptozotocin-induced diabetes, suggesting that PAR-1 deficiency might even aggravate diabetic nephropathy. Our finding that PAR-1 deficiency limits diabetic nephropathy seems to contradict these data. This may likely be a result of biased agonism^15^, a term used to describe a phenomenon in which one receptor, in this case, PAR-1, can be cleaved and activated by different agonists leading to different intracellular signaling pathways, and subsequently, different physiological processes. Indeed, APC-induced PAR-1 activation leads to a decrease in podocyte apoptosis^13^, whereas thrombin-induced PAR-1 activation leads to an increase in podocyte apoptosis^16^. Importantly, apart from podocytes, PAR-1 is expressed on mesangial cells and glomerular endothelial cells. Therefore, cell type-dependent PAR-1 signaling might also explain why a complete PAR-1 knockout protects against diabetic nephropathy, despite (APC-dependent) PAR-1-mediated prevention of podocyte loss^13^. Moreover, the effect of PAR-1 on the development of diabetic nephropathy may be dependent on the (range of) agonist(s) expressed during diabetes.

Irrespective of the actual agonist activating PAR-1 during diabetes, we show here that the net effect of PAR-1 during diabetes is detrimental. The diminished nephropathy observed in PAR-1 deficient diabetic mice was histologically characterized by reduced expansion of the mesangial area and diminished tubular atrophy. As it is well known that the degree of proteinuria strongly correlates with tubular atrophy in progressive kidney diseases^17^,^18^, reduced tubular atrophy in PAR-1 deficient mice is likely secondary to diminished glomerular damage and consequent protein leakage. Alternatively, tubular injury may induce glomerular damage (as excellently reviewed by Gilbert and Cooper^19^). Indeed, exposure of proximal tubular cells to glucose induces profibrotic responses such as production of TGFβ and ECM proteins. More recently, it was shown that Sirt1 production in proximal tubules may maintain podocyte function thereby limiting albuminuria during diabetes^20^. As glucose did not induce PAR-1 expression on proximal tubular epithelial cells and as we did not observe an effect of PAR-1 stimulation on proliferation of these tubular epithelial cells, we hypothesize that PAR-1 mainly acts upon mesangial cells. Importantly however, PAR-1 activation by thrombin stimulates the expression of TGF-β with subsequent extracellular matrix production by proximal tubular epithelial cells^21^ which would suggest PAR-1 activation on tubular epithelial cells may potentiate tubular atrophy directly. Intriguingly however, these latter responses seem context dependent, as thrombin down-regulates the TGF-β-mediated expression of ECM proteins in the presence of APC^22^. The *in vivo* role of PAR-1 on tubular atrophy needs consequently to be established in more specific models inducing primary tubular damage without glomerular injury although such models do not mimic tubular atrophy in diabetic nephropathy.

Interestingly, thrombin-induced PAR-1 signaling leads to increased mesangial proliferation *in vitro*. In line, we observed a decreased number of proliferating cells in PAR-1 deficient mice, suggesting that PAR-1 mediated signaling did indeed contribute to mesangial expansion in diabetic nephropathy. Similarly, fibronectin deposition was increased after thrombin-mediated PAR-1 signaling in mesangial cells *in vitro* whereas it was reduced in glomeruli of diabetic PAR-1 deficient mice. Interestingly, as opposed to fibronectin deposition, collagen IV and αSMA were not significantly reduced in PAR-1 deficient mice, while PAR-1 activation, on mesangial cells *in vitro*, did lead to increased production of these extracellular matrix proteins (Supplementary Fig. S2). The lack of an *in vivo* increase in collagen IV may be caused by the fact that the glomerular density of collagen IV has been shown to decrease during diabetic nephropathy due to enlargement of the overall mesangial area^23^. The PAR-1-induced production of collagen IV may thus be counterbalanced by this phenomenon. Most likely, we did not observe a decrease in αSMA levels in PAR-1 deficient mice because αSMA levels are already low in diabetic wild type glomeruli.

Thrombin-induced PAR-1 activation was recently shown to promote fibronectin secretion by mesenchymal stem cells via ERK1/2 activation^24^. In line with these data, we show that inhibition of the MEK-ERK pathway prevents both PAR-1-induced proliferation and fibronectin production in mesangial cells. PAR-1-induced ERK seems dependent on the Src-MEK pathway, a well-known pathway downstream of PAR-1^25^, as indeed the inhibition of these signaling molecules also prevent PAR-1-induced proliferation and fibronectin production. Intriguingly, we show that TGFβ receptor (TGFBR) inhibitors also prevent PAR-1-induced fibronectin production by MES13 cells despite the fact the cells do not seem to produce TGFβ in response to PAR-1 activation. As G-protein coupled receptors in general, and PAR-1 in particular, transactivation of the TGFβ receptor has recently been described^26^,^27^,^28^, it is tempting to speculate that PAR-1-mediated TGFBR transactivation leads to fibronectin expression in MES13 cells. Transactivation may subsequently lead to non-canonical TGFβ signaling^29^, resulting in MEK and ERK activation thereby explaining the inhibitor effect of MEK inhibition on fibronectin production in our study. Alternatively, as ERK potentiates SMAD phosphorylation^30^, PAR-1-dependent ERK activation may induce fibronectin production by potentiating canonical TGFβ signaling upon transactivation of the TGFBR.

The key role of PAR-1 in the development of diabetic nephropathy suggests that PAR-1 is indeed a potentially interesting target to pursue, for the prevention of diabetic nephropathy. Notably, the formation of thrombin, the prototypical PAR-1 agonist, is increased in patients with diabetes^31^. Surprisingly, until now, large clinical trials studying the effect of thrombin lowering agents, such as sulodexide, in diabetic patients, fail to show (convincing) renal protective effects^32^,^33^,^34^. We therefore hypothesize that alternative PAR-1 agonists may be responsible for driving diabetic nephropathy, and the identification of these PAR-1 agonists is currently under investigation. Apart from the well-known PAR-1 agonists, thrombin and APC, several other proteases, including MMP-1^35^, MMP-13^36^, granzyme K^37^, kallikrein-1^38^ and -6^39^, kallikrein-related peptidase 4^40^, proteinase 3^41^, neutrophil elastase^41^, and PRSS3^42^ have been described to activate PAR-1 in different pathological settings. In the setting of diabetic nephropathy, kallikrein-1 limits renal injury as evident from increased albuminuria in diabetic kallikrein-1 deficient mice as compared to wild type controls^43^. Further studies investigating the agonist(s) expressed in the kidney during diabetes are needed to elucidate which agonist(s) are responsible for driving diabetic nephropathy.

Although the PAR-1-dependent mechanisms driving diabetic nephropathy are complex and might be both cell type and agonist dependent, the most important observation of our study is that PAR-1 aggravates the development of diabetic nephropathy, with a net nephroprotective effect of PAR-1 deficiency. We thus provide proof of principle for a detrimental role for PAR-1 in diabetic nephropathy which suggests that PAR-1 inhibition could be a potential strategy for future therapy. However, subsequent experiments addressing pharmacological inhibition of PAR-1 in preclinical models should elucidate whether PAR-1 inhibition indeed has clinical potential.

## Methods

### Mice

Heterozygous PAR-1 deficient mice, developed on a C57Bl/6 background were purchased from The Jackson Laboratory (Bar Harbor, ME, USA)^44^. Animals were intercrossed to obtain homozygous PAR-1 deficient mice, as described before^45^. Wild type C57BL/6 mice were purchased from Charles River (Maastricht, the Netherlands). All experiments were approved by the Institutional Animal Care and Use Committee of the University of Amsterdam. All mice were maintained according to institutional guidelines. Animal procedures were carried out in compliance with the Institutional Standards for Humane Care and Use of Laboratory Animals of the Academic Medical Center. The Animal Care and Use Committee of the Academic Medical Center approved all experiments.

### Experimental diabetic nephropathy model

Eight to twelve week-old male wild type and PAR-1 deficient mice (8 per group) were injected with streptozotocin (50 mg/kg body weight) for 5 consecutive days to induce diabetes. Eight age-matched, untreated wild type and PAR-1 deficient mice served as a non-diabetic control groups. Three months after streptozotocin injections, mice were sacrificed, and blood, urine and kidneys were harvested for further analysis. Blood glucose levels were measured from tail vein blood using a Bayer Contour glucose meter. Plasma cystatin C (R&D systems) and urine albumin (Bethyl laboratories) levels were determined by ELISA according to the manufacturer’s instructions. Urine creatinine levels were determined using an enzymatic mouse creatinine assay kit (CrystalChem), according to the manufacturer’s instructions.

### (Immuno)histopathology

Formalin-fixed, paraffin embedded, kidney sections were subjected to periodic acid–Schiff–diastase (PAS-D) and Masson’s Trichrome staining, following routine procedures. The extent of glomerular injury was determined by two independent observers in a blinded fashion. To quantify glomerular injury, 50 glomeruli per mouse, were scored as either normal or deviated. Glomeruli were scored as deviated when mesangial expansion was apparent as clusters of >3 mesangial cells. Glomerular extracellular matrix deposition was scored semi-quantitatively using the following score: 0: no apparent matrix deposition; 1: up to 20% matrix deposition; 2: 20–40% matrix deposition; 3: more than 40% matrix deposition.

Proliferative and apoptotic cells were detected using rabbit-anti-Ki67 (1:500; #RM-9106; Lab Vision) and rabbit-anti-cleaved caspase-3 (1:200; #9661S; Cell Signaling Technologies) antibodies. Glomerular extracellular matrix accumulation and mesangial activation were determined using rabbit-anti-collagen type I (1:400; GTX41286; GeneTex), rabbit-anti-collagen type IV (1:1000; ab6586; Abcam), goat-anti-fibronectin (1:500; sc-6953; Santa Cruz Biotechnology) and mouse-anti-smooth-muscle-α-actin (α-SMA) (1:200; sc-32251; Santa Cruz Biotechnology) antibodies as described before^7^. In short, paraffin embedded slides were deparaffinized, and endogenous peroxidases were inhibited by 15 minutes incubation in 0,3% H_2_O_2_ at room temperature. Slides were boiled in citrate buffer (pH6.0) for 10 min, blocked with normal goat serum or Ultra V block (Thermo Scientific, Runcorn, UK) for 30 min, and incubated overnight with the primary antibody. Slides were incubated with Powervision PolyHRP-anti-rabbit IgG (DPVR-55HRP; Immunologic), HRP conjugated rabbit-anti-goat IgG (P0160; Dako), or HRP conjugated goat-anti-mouse IgG (P0447; DAKO) for 30 min at room temperature, visualized with DAB (BS04-999; Immunologic) and counterstained using haematoxylin. Slides incubated without the primary antibody were used as negative controls to exclude nonspecific binding of the secondary antibody. Pictures were taken at 20 times magnification using a Leica DM5000B microscope equipped with a Leica DFC500 camera and Image Pro Plus software (vs 5.02; Media Cybernatics). The Collagen I, Collagen IV, fibronectin, and α-SMA positive areas (expressed as a percentage) was determined, per glomerulus (expressing the density of ECM) using ImageJ software (U.S. National Institutes of Health, Bethesda, MD, USA) in 25 glomeruli per mouse. The number of Ki67 or caspase-3 positive cells was counted by two independent researchers and expressed as the amount of positive cells per 50 glomeruli.

### RNA isolation and RT qPCR

For gene expression analysis, mRNA was isolated from kidney homogenates or cultured cells using TriReagent isolation reagent (#11667165001; Roche Diagnostics) according to the manufacturers recommendations. All mRNA samples were quantified by spectrophotometry and stored at −80 °C until further analysis. 1 μg of mRNA was treated with DNAse using the RQ1 DNAse kit (M6101, Promega, Madison, WI, USA) and subsequently converted to cDNA using M-MLV reverse transcriptase (M1705, Promega, Madison, WI, USA) and random hexamer primers (#SO142, Fisher scientific, Landsmeer, the Netherlands) according to the manufacturers recommendations. qPCR and subsequent analysis were performed using a Roche lightcycler with SYBR green PCR master mix (#04707516001; Roche, Almere, the Netherlands) on a Lightcycler 480 machine and corresponding software (Software release 1.5.0 (1.5.0.39), Roche, Almere, the Netherlands). Expression levels were normalized using the average expression levels of GAPDH and TBP. The following primer sequences were used: mPAR-1 forward: 5′-GTTGATCGTTTCCACGGTCT-3′reverse: 5′-ACGCAGAGGAGGTAAGCAAA-3′; mTBP forward: 5′-GGAGAATCATGGACCAGAACA-3′ reverse: 5′-GATGGGAATTCCAGGAGTCA-3′; hPAR-1 forward: 5′-GCAGGCCAGAATCAAAAGCAACAAATGC-3′ reverse: 5′-TCCTCATCCTCCCAAAATGGTTCA-3′; hTBP forward: 5′-ATCCCAAGCGGTTTGCTGC-3′ reverse: 5′-ACTGTTCTTCACTCTTGGCTC-3′.

### Cell culture and stimulation

Mouse mesangial cells (SV40 MES13; CRL-1927 ATCC) were cultured according to the recommended protocol using a 3:1 mixture of Dulbecco’s Modified Eagle’s Medium containing 1 g/L glucose with Ham’s-F12 medium, supplemented with heat inactivated fetal calf serum (5%), 100 U/ml penicillin, 100 μg/ml streptomycin, and 2 mM L-glutamine. Human kidney 2 (HK2) proximal tubular epithelial cells (PTECs) were cultured according to the recommended protocol using a 1:1 mixture of Dulbecco’s Modified Eagle’s Medium containing 1 g/L glucose with Ham’s-F12 medium, supplemented with heat inactivated calf serum (10%), 100 U/ml penicillin, 100 μg/ml streptomycin, 2 mM L-glutamine, 5 μg/ml insulin, 5 μg/ml transferrin, 5 μg/ml selenite, 20 ng/ml Tri-iodo-thyrionine, 50 ng/ml Hydrocortisone, and 5 ng/ml Prostaglandine E1. MES13 and HK-2 cells were cultured at 37 °C in an atmosphere of 5% CO_2_. All cells were serum starved in medium containing 1 g/L (LG) glucose for at least 4 hours before stimulation with 1 U/ml thrombin (sigma; St Louis, Missouri, USA) or 100 μM PAR-1 agonist peptide (PAR-1-AP; H-SFLLRN-NH2; Biochem, Shanghai, China) in HG, LG medium, or LG medium supplemented with 20 mM mannitol (as indicated). When indicated cells were pretreated with 10 μM PAR-1 pepducin (P1pal12; palmitate-RCLSSSAVANRS-NH2; Biochem), 20 nM mTOR inhibitor (rapomycin), 200 nM Src inhibitor (PP1, BioMal, UK), 10 μM p38 inhibitor (SB203580, LC laboratories, MA, USA), 1 μM PKC inhibitor (Chelerythrine, Sigma, The Netherlands), 10 μM MEK inhibitor (U0126), or 10 μM TGFb inhibitors (SB-431542, Sigma, The Netherlands and LY-215729, Axon Medchem, The Netherlands).

### MTT assay

Cells were seeded at a density of 4000 cells/well in 96 wells plates. After stimulation with thrombin or PAR-1 agonist peptide for 24 hours, 0.5 mg/ml MTT was added to the culture medium. After 1 hour incubation at 37 °C, cells were lysed with DMSO and OD_570_ was measured using a microplate reader (Synergy HT, BioTek).

### Western Blot

Cells were seeded at a density of 20000 cells/well in 24 wells plates. After stimulation for 24 hours, cells were washed in ice cold PBS and lysed in Laemmli buffer. Cell lysates were separated on 8% SDS-PAGE gel and transferred onto Immobulin-FL membranes (Millipore) using routine procedures. Membranes were blocked for 1 hour at room temperature in 5% bovine serum albumin (BSA) in TBS + 0,1% tween-20 (TBS-T) and subsequently incubated with the following primary antibodies, diluted in TBS-T: mouse-anti-tubulin 1:2500 (Santa Cruz; sc-23948); mouse-anti-GAPDH 1:1000 (Santa Cruz; sc-32233); mouse-anti-b-actin 1:1000 (Santa Cruz; sc-81178); rabbit-anti-collagen IV 1:1000 (abcam ab6586//Sigma SAB4500309); goat-anti-fibronectin 1:1000 (Santa Cruz; sc-6953); mouse-anti-α-SMA 1:1000 (Santa Cruz; sc-32251); rabbit-anti-phospho-p44/42 MAPK 1:1000 (Cell Signaling; #9101). After overnight incubation, the membranes were washed with TBS-T and incubated 1 hour at room temperature with horseradish peroxidase (HRP)-conjugated (1:1000, DakoCytomation, Glostrup, Denmark) or IRDye (700-donkey-anti-rabbit, IRDye 800-donkey-anti-rabbit, or IRDye 800-donkey-anti-mouse, 1:5000) secondary antibodies, diluted 1:5000 in TBS-T. Membranes were washed in TBS and imaged using Lumi-Light (12015200001; Roche, Basel, Switzerland) on an ImageQuant LAS 4000 biomolecular imager (GE Healthcare, Zeist, the Netherlands), or using an Odyssey IR Imager (LI-COR Bioscience, Westburg b.v., Leusden, the Netherlands).

### Statistics

All values are expressed as mean ± SEM. All groups were tested for normality and for outliers using the D’Agostino-Pearson omnibus normality test. Detected outliers were excluded from analysis. Differences between two groups were analysed using a *t*-test if data were normally distributed, or a Mann-Whitney *U*-test for non-parametric data. Multiple comparisons were analysed using one-way-ANOVA analysis or Kruksal-Wallis test (for nonparametric values), followed by Bonferroni’s or Dunns multiple comparison tests, respectively. All analyses were performed using GraphPad Prism version 5.01.

## Additional Information

**How to cite this article**: Waasdorp, M. *et al*. Protease-activated receptor-1 deficiency protects against streptozotocin-induced diabetic nephropathy in mice. *Sci. Rep*. **6**, 33030; doi: 10.1038/srep33030 (2016).

## Supplementary Material

Supplementary Information
