# Supplementary material for: Protease-activated receptor-1 deficiency protects against streptozotocin-induced diabetic nephropathy in mice
Source: Sci Rep. 2016 Sep 13;6:33030. doi: 10.1038/srep33030 (PMC5020504; doi:10.1038/srep33030)
Supplement: Supplementary Information [file srep33030-s1.pdf]

Protease-activated receptor-1 deficiency protects against streptozotocin-induced diabetic nephropathy in mice by diminishing mesangial expansion.

Maaike Waasdorp[1, †, *], JanWillem Duitman[1, †], Sandrine Florquin[2] and C. Arnold Spek[1]

[1]Center for Experimental and Molecular Medicine, Academic Medical Center, Amsterdam, 1105 AZ, The Netherlands.

[2]Department of Pathology, Academic Medical Center, Amsterdam, 1105 AZ, The Netherlands.

*Corresponding author: Maaike Waasdorp, Center for Experimental and Molecular Medicine, Academic Medical Center, Meibergdreef 9, 1105 AZ Amsterdam, the Netherlands. Phone: +31 20 56 65 247; E-mail: M.Waasdorp@amc.uva.nl.

[†] These authors contributed equally.

Supplementary Figure S1

Supplementary Figure S2

Supplementary Figure S1

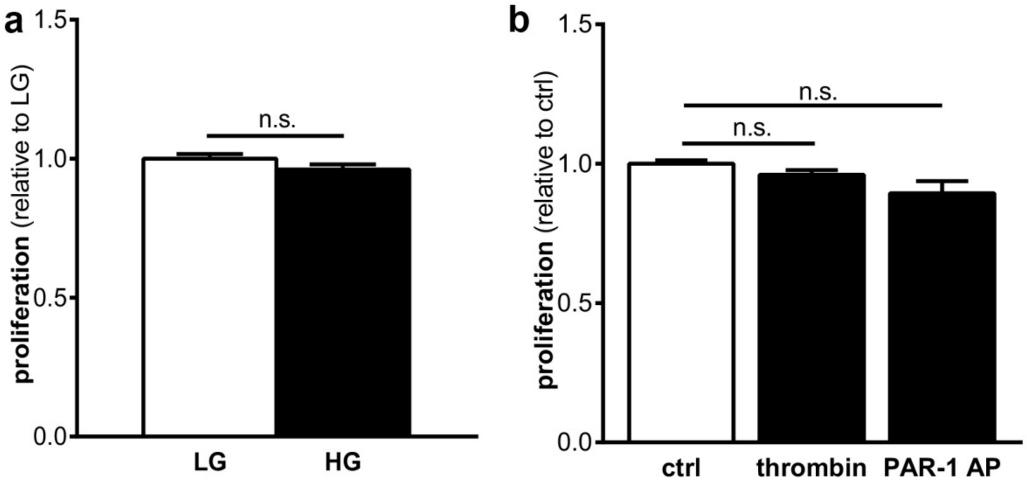

Supplementary Figure S1. Proliferation of HK2 cells after high glucose or PAR-1 stimulation is unaltered. (**a**) Proliferation of HK2 cells after 24h of stimulation with 25 mM glucose. (**b**) Proliferation of MES13 cells after 24h stimulation with thrombin (1 U/ml) or PAR1-AP (100 μM). Indicated is the mean ± SEM, n=3 individual experiments. One-way ANOVA with Bonferroni post-hoc analysis was used.

Supplementary Figure S2.

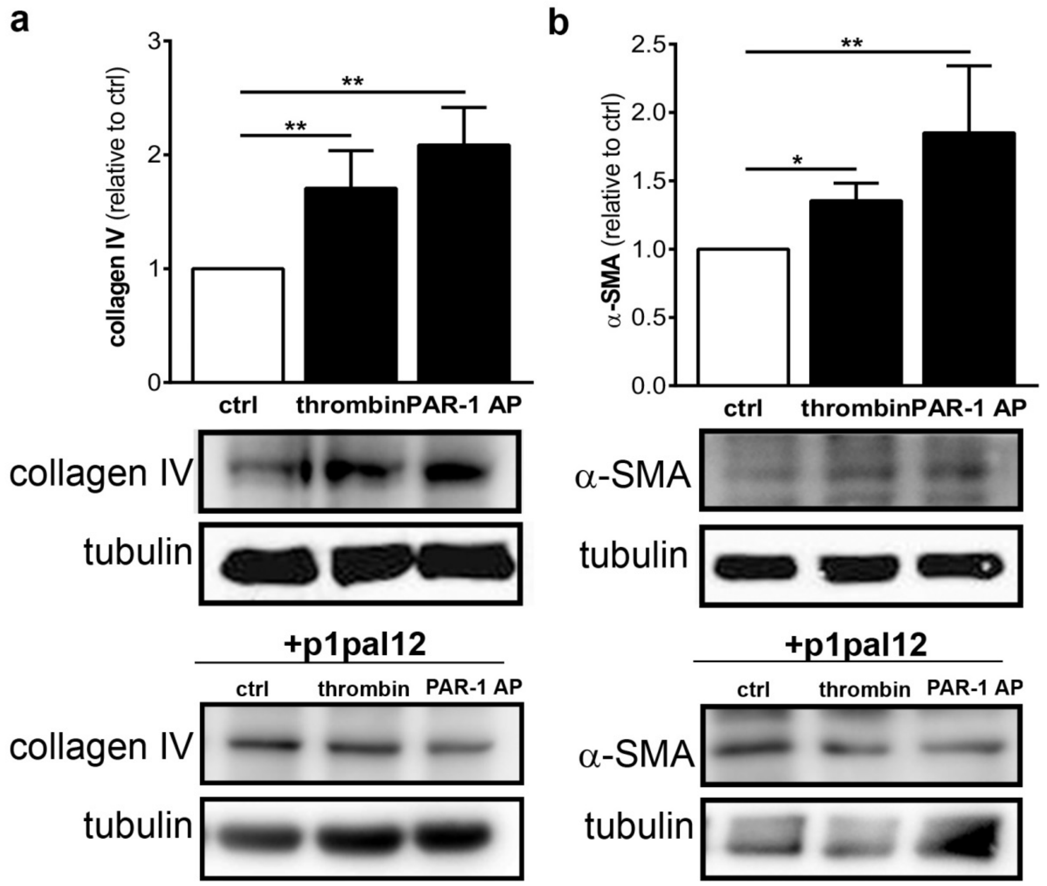

Supplementary Figure S2. PAR-1 stimulation leads to increased collagen IV and aSMA production in MES13 cells. Western blot (lower panel) and quantification (upper panel) of collagen IV (**a**) and αSMA levels in MES13 whole cell lysates after 24h stimulation with thrombin (1 U/ml) or PAR1-AP (100 μM), with and without p1pal12 pretreatment. Indicated is the mean ± SEM, n=3 individual experiments. One-way ANOVA with Bonferroni post-hoc analysis was used *: $p<0.05$; **: $p<0.01$.
